# Exploring Body Image Dissatisfaction and Psychiatric Co-morbidities in Rural India: A Comprehensive Review

**DOI:** 10.7759/cureus.53123

**Published:** 2024-01-28

**Authors:** Nayan Sinha, Pradeep S Patil, Imyarila Longkumer, Yatika Chadha

**Affiliations:** 1 Psychiatry, Jawaharlal Nehru Medical College, Datta Meghe Institute of Higher Education & Research, Wardha, IND

**Keywords:** intervention strategies, sociocultural factors, psychiatric co-morbidities, mental health, rural india, body image dissatisfaction

## Abstract

This comprehensive review examines the intricate landscape of body image dissatisfaction (BID) in rural India, shedding light on the multifaceted factors influencing individual perceptions and societal expectations. Delving into cultural nuances, economic disparities, and gender-specific experiences, the study highlights the pervasive nature of BID across diverse age groups. Unveiling the complex interplay between BID and psychiatric co-morbidities, such as depression and anxiety, underscores the urgency of integrated mental health interventions. The review concludes with a resounding call to action for policymakers, healthcare professionals, and communities. It advocates for culturally sensitive mental health policies, targeted healthcare training, and community-driven initiatives to foster environments conducive to positive body image and mental well-being. By acknowledging these challenges and committing to collaborative solutions, this review aims to contribute to developing comprehensive strategies that address BID in rural India and pave the way for healthier and more resilient communities.

## Introduction and background

Body image dissatisfaction (BID) is a complex psychological phenomenon encompassing an individual's thoughts, feelings, and perceptions regarding their physical appearance. It extends beyond physical attributes, delving into self-esteem, identity, and societal expectations. Understanding the nuanced nature of BID is crucial for comprehending its impact on mental health and developing targeted interventions [1]. Focusing on rural India brings a distinctive perspective to studying BID. Rural communities are often characterized by distinct cultural practices, traditional beauty standards, and limited access to resources compared to urban counterparts. Exploring BID in this context provides insights into how socio-cultural factors intersect with perceptions of body image, shedding light on unique challenges and opportunities for intervention [2].

The primary objective of this review is to provide a comprehensive examination of the prevalence, contributing factors, and psychiatric co-morbidities associated with BID in rural India. This review aims to offer a nuanced understanding of the dynamics at play by synthesizing existing literature and research findings. Additionally, the review will explore potential interventions and suggest directions for future research, ultimately contributing to a more holistic approach to mental health in rural settings.

## Review

Prevalence of BID in rural India

Cultural Factors Influencing Body Image

Traditional beauty standards: BID in rural India is intricately shaped by a multifaceted interplay of psychological, physical, and cultural factors. Notably, traditional beauty standards significantly influence the definition of ideal body image. Extensive research has demonstrated that in the Indian cultural context, beauty standards are deeply entrenched in societal values, associating attributes like success and power with specific body images. This creates a pervasive pressure to conform to these ideals, with familial and peer criticism exacerbating BID. Moreover, the impact of media messages and the internalization of societal expectations emanating from family, peers, and media stand out as significant contributors to BID in the Indian milieu [3,4]. Traditional beauty standards in India, valuing plumpness and equating it with beauty, reveal a departure from the indigenous norm of attractiveness and prosperity, indicative of the globalized influences on beauty standards [4]. This underscores the complexity and multifaceted nature of the influence of cultural factors on BID in rural India. Despite existing studies on the prevalence of BID and its correlates in the Indian context, there exists a critical need for further research to discern the specific cultural factors shaping BID in rural India and to devise precise interventions to address this burgeoning concern [3,4].

Influence of media and globalization: The impact of exposure to slender body images, propagated as societal ideals through both local and global media, has been identified as a significant factor contributing to heightened disturbances in body perception among women in both metropolitan and rural settings [5]. The pervasive influence of thin-ideal media representations is particularly noteworthy, as research indicates that the internalization of such images correlates with an escalation in body dissatisfaction and a simultaneous decline in self-esteem among young Indian women [6]. A comprehensive investigation into the interplay between media pressure, internalization, and various aspects of body image has revealed positive associations between body dissatisfaction, disordered eating, body mass, and media influence. Notably, media internalization and pressure, in conjunction with body mass index (BMI), emerged as substantial predictors of body dissatisfaction among the youth in India [4]. Another dimension explored in this research is the deviation from traditional beauty standards in India. The study uncovered that prevailing beauty norms, where plumpness is highly valued and often equated with beauty, deviate from indigenous standards of attractiveness and prosperity, indicating the impact of globalization on beauty ideals [4]. Despite existing studies that delve into dissatisfaction levels and fat-phobic attitudes within the Indian population, research on South Asian ethnicity remains limited, and there is a noticeable underrepresentation of men from ethnic minorities in available studies [4]. The substantial influence of media and globalization on BID in rural India is evident, with exposure to slender body ideals, media-induced pressure, and internalization of these ideals collectively contributing to heightened levels of body dissatisfaction. Addressing this growing concern necessitates further research to understand the cultural factors influencing BID in rural India. Additionally, there is a pressing need to formulate targeted interventions tailored to the unique dynamics at play to mitigate the impact of media and globalization on BID [4-6].

Gender Differences in BID

Gender differences in BID have been extensively documented, consistently revealing higher levels of BID in women compared to men. However, a nuanced landscape exists as some studies present contrary findings or propose variations based on contextual factors or measurement methodologies. The general trend indicates that women consistently report elevated BID levels compared to men [7-9]. Men, on the other hand, tend to express lower dissatisfaction with their bodies, perceiving themselves as more attractive and less influenced by BID than their female counterparts [8]. The manifestation of thinness-oriented BID tends to be more pronounced in women, whereas muscularity-oriented BID is typically heightened in men [10]. While some studies find no significant gender differences in BID [7], others report higher levels of weight dissatisfaction in men compared to women [7]. Despite the consensus that women generally experience higher BID levels than men, specific research highlights conflicting results, suggesting potential variations dependent on specific contexts or measurement criteria. Further investigation is crucial to comprehensively elucidate the contributing factors to these gender differences. Moreover, there is a pressing need to develop targeted interventions addressing BID in both men and women, considering the intricacies and diversities within these experiences [3,8-11].

Age Groups Affected

Body image concerns transcend age, gender, and cultural boundaries, impacting individuals universally. Nevertheless, BID varies across different age groups. Research indicates that the levels of body dissatisfaction may not significantly differ across ages in women, contrasting findings that suggest potential changes in BID across age groups in men [11]. Notably, the significance of appearance appears to diminish with age in women, while in men, it tends to remain relatively stable over time [11]. Studies underscore the correlation between body appreciation and low levels of body dissatisfaction in both men and women [11]. The prevalence of body dissatisfaction is particularly noteworthy among adolescents in low- and middle-income countries, including India [12]. A study conducted in rural Tamil Nadu, India, specifically aimed to compare BID among adolescent boys and girls. Still, the search results should have provided specific findings [13]. Acknowledging that body image issues affect individuals of all ages, the nuances of this experience demand consideration of age and gender dynamics. While specific studies propose that body dissatisfaction remains consistent across age in women, others suggest potential changes across age in men. To comprehensively address this issue, further research must explore the contributing factors to age-related differences in BID. Developing targeted interventions tailored to different age groups is crucial for promoting positive body image across the lifespan [11-14].

Factors contributing to BID

Sociocultural Factors

Social pressure and expectations: Sociocultural factors, encompassing media influence, family dynamics, peer pressure, and cultural norms, have been identified as pivotal contributors to the development of BID [4,15,16]. The internalization of a slender ideal, awareness of societal pressures to adhere to this standard, and perceived expectations of attaining such an ideal are recognized as fundamental constructs associated with body dissatisfaction [15]. Additionally, social pressure and expectations, such as the imperative to conform to cultural norms of attractiveness and prosperity, have been implicated in the prevalence of body dissatisfaction within traditional societies, exemplified by rural India [4]. These factors positively correlate with body dissatisfaction, disordered eating, and BMI [4,15,17]. Further research is imperative to delve into the intricate interplay between sociocultural factors and BID in rural India, aiming to develop effective interventions to address this burgeoning concern.

Influence of family and peers: The impact of family dynamics on children's body image is underscored by research demonstrating that parental endorsement of weight loss activities and adherence to diets can significantly shape children's perceptions of their bodies [18]. Conversely, a lack of social support from parents has been identified as a correlating factor with body dissatisfaction in young adolescents [19]. Within peer relationships, discussions centered around beauty and appearance are pivotal in shaping adolescents' feelings and perceptions regarding their bodies [18]. Peer ridicule has emerged as a crucial predictor of dissatisfaction with one's physical appearance [20]. Media also exerts a substantial influence on shaping body image, as exposure to images and articles promoting an "ideal body image" has been linked to negative impacts on body image cognition [20]. The internalization of a slender ideal and the pressure to conform to societal expectations associated with this ideal are recognized as significant constructs related to body dissatisfaction [18,19]. Additionally, cultural and societal norms further contribute to the landscape of body image satisfaction. Notably, research highlights that girls as young as five years old may start expressing dissatisfaction with their bodies. At the same time, boys tend to articulate body dissatisfaction only in their teenage years, influenced by factors such as masculinity and peer pressure [18]. The interplay of family, peer, and media influences assumes critical roles in shaping BID. However, the intricacies of the interactions among these factors and their collective impact on BID warrant further exploration. Consequently, additional research is essential to unravel the complexities of this interplay and to develop effective interventions aimed at addressing the escalating concern of BID.

Economic Factors

Access to beauty products and services: Access to beauty products and services is subject to the sway of economic factors, encompassing challenges such as inflation, disruptions in the supply chain, and fluctuations in interest rates [21]. The beauty industry, anticipated to burgeon to approximately $580 billion by 2027, is on a trajectory of sustained annual growth at 6 percent [22]. Notably, the luxury and ultraluxury beauty market, presently valued at around $20 billion, holds the potential to double its worth, reaching approximately $40 billion by 2027 [22]. Economic conditions also wield influence over the demand for cosmetics, with macroeconomic factors such as regular pricing, relative brand pricing, and reference pricing intricately shaping the pricing dynamics of cosmetic products [23]. Furthermore, the cost of cosmetic surgery procedures is intricately correlated with local economic factors, including population size, cost-of-living index, and real estate considerations [23]. In sum, economic factors significantly impact the accessibility and affordability of beauty products and services, consequently influencing body image satisfaction and self-esteem.

Impact of socioeconomic status on body image: Numerous studies have delved into the intricate relationship between socioeconomic status (SES) and body image, revealing multifaceted dynamics. A study examining users of hook-up apps uncovered a positive correlation between higher SES and elevated body appreciation coupled with diminished body dissatisfaction [24]. Investigations spanning children and adolescents aged 6 to 19 unveiled that SES, age, weight, and gender exert a discernible influence on body image and weight control practices, with higher SES potentially affording a protective effect on the body image of young individuals [25]. Additional research demonstrated a connection between greater stature in males and higher family income, while heightened obesity in women was associated with lower family income [26]. In the context of body weight dissatisfaction among men and women across different SES groups, a study revealed that higher SES correlated with an increased likelihood of dissatisfaction with body weight among preobese men [27]. These collective findings underscore the nuanced impact of SES on body image perceptions and dissatisfaction. While higher SES generally aligns with more positive body image perceptions, this relationship proves complex and subject to variations based on gender and other influencing factors. The call for further research persists, urging a comprehensive understanding of the complete spectrum of SES effects on body image and the identification of potential interventions to address BID across diverse socioeconomic groups.

Health and Nutritional Factors

Malnutrition and its effect on body perception: Malnutrition is a significant determinant of body perception, with physical starvation and malnourishment contributing to adverse body image and negative self-perceptions [28]. This impact extends to body weight status, particularly evident among children residing in welfare homes who confront malnutrition challenges [29]. The repercussions of malnutrition go beyond physical health, as it directly affects cognitive functions, reasoning abilities, intellectual capacity, and judgment, collectively fostering negative body image perceptions [28,30]. Furthermore, an intriguing finding from a study highlights that higher socioeconomic status is associated with an increased likelihood of dissatisfaction with body weight among preobese men, underlining the role of socioeconomic factors in the realm of BID [27]. Consequently, socioeconomic status is a pivotal factor in shaping body image perceptions. In light of these insights, addressing malnutrition must be accorded priority in treatment approaches to holistically improve overall body image perceptions [28]. Recognizing the intricate interplay between nutritional well-being, socioeconomic factors, and body image is essential for developing comprehensive interventions to foster positive body perceptions and mitigate malnutrition's impact on individual well-being.

Influence of physical health on body image: Research underscores a robust correlation between physical health and body image, revealing a reciprocal relationship where positive body image aligns with enhanced physical well-being. Individuals with a positive body image tend to exhibit healthier lifestyle choices, such as increased engagement in physical activity, reduced smoking and alcohol consumption, and fewer negative eating habits [31]. Conversely, malnutrition and physical starvation contribute to a detrimental impact on body image, fostering negative perceptions [32]. A healthy relationship with physical activity is pivotal, emphasizing the importance of regular, enjoyable exercise. Individuals harboring positive body image perceptions are more inclined to participate in physical activities than those grappling with negative body image [32]. Various factors contribute to the development of negative body image, encompassing childhood experiences of appearance or weight-related teasing, family, and friends promoting dieting and expressing body dissatisfaction, cultural tendencies to judge individuals based on appearance, and peer pressure among girls and women to conform to slim ideals, engage in diets, exercise, and engage in constant comparisons with others [33]. The intertwining of physical health and body image underscores the need for a comprehensive approach to health and well-being. Addressing both aspects is essential to improve overall health, emphasizing the significance of interventions that promote positive body image and foster healthy lifestyle choices. Recognizing these interconnections enhances our understanding of the holistic nature of well-being and guides efforts to cultivate a positive and supportive environment for individuals to thrive physically and mentally.

Psychiatric co-morbidities associated with BID

Depression and Anxiety

BID is linked to psychiatric co-morbidities such as depression and anxiety [34-36]. A study conducted on psychiatric outpatients in Singapore revealed a high comorbidity of depression and anxiety stemming from dissatisfaction with body appearance [34]. A systematic review and meta-analysis identified statistically significant associations between body satisfaction and anxiety (0.40, 95% CI) in men [35]. In Swedish adolescents and young female adults, body dissatisfaction correlates with both depression and anxiety [36]. A study involving individuals with obesity found that perceived stress acts as a mediator in the relationship between body image and depressive symptoms [37]. A cross-sectional study on Indian obese school children established a connection between BID, depression, and health-related quality of life [38]. The repercussions of these associations can detrimentally impact mental health and overall well-being [34-36]. Further research is imperative to elucidate the causal relationships between these factors and to formulate targeted interventions addressing BID and its concomitant psychiatric issues [34-36].

Eating Disorders

Patients with eating disorders commonly experience various psychiatric co-morbidities, including depression, anxiety disorder, obsessive-compulsive disorder, substance abuse, attention-deficit hyperactivity disorders, and personality disorders [39,40]. Moreover, individuals grappling with BID are at an increased risk of encountering depression and anxiety and engaging in disordered eating behaviors [34]. Notably, a study revealed that individuals concurrently dealing with body dysmorphic disorder and eating disorders exhibited more pronounced body image disturbances and sought mental health treatment more frequently than those without a comorbid eating disorder [41]. These insights underscore the intricate interconnections between BID, eating disorders, and a spectrum of psychiatric co-morbidities, underscoring the necessity of addressing these intertwined conditions concurrently in clinical practice.

Substance Abuse

Current research underscores a direct correlation between substance abuse and BID [42]. Individuals grappling with poor body image are more inclined to engage in drug or alcohol abuse, and those contending with eating disorders and negative body image are particularly prone to using substances as a coping mechanism [42]. While specific information on psychiatric co-morbidities linked to both substance abuse and BID is not readily available in the current search results, it is noteworthy that individuals with eating disorders, often intertwined with BID, commonly experience various psychiatric co-morbidities such as depression, anxiety disorder, obsessive-compulsive disorder, and personality disorders [39-41,43]. This suggests a broader context wherein the complexities of substance abuse, BID, and associated psychiatric co-morbidities warrant further investigation for a comprehensive understanding of the intricate relationships involved.

Challenges in addressing BID in rural India

Limited Mental Health Resources

Lack of mental health professionals: In rural areas, there is a significant dearth of trained mental health professionals, including psychologists and psychiatrists. This scarcity poses a considerable obstacle for individuals seeking assistance, as the limited availability of specialized professionals hampers access to appropriate mental health services [4,13]. The shortage of such professionals exacerbates the challenge of effectively addressing concerns related to BID and other mental health issues in rural communities.

Limited awareness: A prevalent issue in rural areas is the insufficient awareness among individuals regarding the signs and symptoms of mental health issues, including BID. This lack of awareness impedes the ability of individuals to recognize their struggles and inhibits them from actively seeking the necessary help and support [4,13]. Addressing this informational gap is crucial for promoting mental health literacy and encouraging proactive engagement with mental health services.

Cultural and societal barriers: Traditional values and cultural norms in rural areas contribute significantly to the stigma associated with mental health issues, including BID. This stigma creates an environment where individuals find it challenging to openly discuss their concerns and seek professional help [4]. The cultural context necessitates culturally sensitive interventions and awareness campaigns to destigmatize mental health issues and encourage a more open dialogue within these communities.

Inadequate funding: Limited funding allocated to mental health services in rural areas results in insufficient resources for the diagnosis, treatment, and support of individuals experiencing BID and other mental health issues [4,13]. This financial constraint underscores the critical need for increased investment in mental health infrastructure in rural regions to enhance the quality and accessibility of mental health care services.

Lack of support systems: Rural areas often lack robust support systems, such as support groups, helplines, and community-based organizations, which can play a pivotal role in assisting individuals struggling with BID and other mental health issues. The absence of such support structures further isolates individuals, hindering their ability to access peer support and guidance [4,13]. Establishing and strengthening community-level support networks is essential for fostering resilience and assisting those in need.

Stigma Around Mental Health

The stigma around mental health is a significant issue in India, which contributes to the underreported prevalence of mental disorders and poor utilization of mental health services [44-47].

Public stigma: In India, the pervasive issue of public stigma significantly contributes to the underreported prevalence of mental disorders, as evidenced by only 7.3% of young people openly acknowledging mental health problems [45]. Stigma manifests as knowledge gaps characterized by ignorance, negative attitudes, prejudice, and discriminatory behaviors [45]. This intricate web of stigma surrounding mental health issues underscores the need for targeted efforts to address misconceptions, change attitudes, and dismantle discriminatory practices.

Fear of judgment: A prevalent barrier to seeking mental health support in India is the pervasive fear of judgment and discrimination faced by individuals with mental health issues [47]. This fear impedes individuals from openly discussing their struggles and seeking the necessary help, creating a significant challenge in raising awareness about mental health and fostering a culture of open dialogue. Mitigating this fear requires concerted efforts to reshape societal perceptions and promote empathy and understanding.

Cultural and societal factors: Stigma surrounding mental health issues is intricately tied to cultural and societal factors, with the extent and nature of stigma varying across cultures. In the Indian context, traditional values and cultural norms contribute significantly to the stigma associated with mental health issues [45]. Addressing this challenge necessitates culturally sensitive interventions that acknowledge and navigate the nuances of Indian society, fostering a more accepting and supportive environment for mental health.

Stigma in rural areas: The impact of stigma is more pronounced in rural areas of India, where the availability of mental health professionals and facilities is limited [46]. Stigma, discrimination, and poor help-seeking behavior converge in rural settings, creating formidable barriers for individuals to access mental health services and support. This exacerbated challenge emphasizes the urgent need for targeted interventions that address stigma at both the community and individual levels in rural areas, ensuring that mental health resources are more accessible and destigmatized.

Efforts to address mental health stigma in India include educating the public about mental health and reducing the stigma associated with mental illness [47]. Involving religious and community leaders in raising awareness about mental health can also help in breaking the stigma surrounding mental health in India [47]. The Indian government has made some efforts to address the issue of mental health. Still, much more needs to be done to ensure that all individuals in need of mental health support can access appropriate services and support [47].

Lack of Awareness and Education on Body Image

Early awareness: The emergence of body image awareness in children and adolescents as young as eight years old underscores the critical importance of early intervention [48]. However, the challenge lies in the availability of resources or curricula tailored to effectively address body image issues, particularly in many schools located in rural areas. Age-appropriate and culturally relevant educational materials are imperative to foster a healthy body image from a young age.

Media literacy and self-esteem: Classroom-based programs focusing on media literacy, self-esteem, and peer influences have effectively promoted a positive body image [47]. Nevertheless, the practical implementation of these programs may be constrained, particularly in rural schools where resources might be limited. Bridging this gap requires concerted efforts to make such programs accessible and applicable in diverse educational settings.

Cultural and ethnic considerations: The universality of body image concerns, even in childhood, includes ethnic minority children and adolescents [47]. However, school-based programs aiming to instill positive body image may need more cultural sensitivity, potentially failing to resonate with the experiences of students from diverse backgrounds. Tailoring interventions to be culturally relevant is essential to ensure inclusivity and effectiveness.

Inadequate health education: Health education in rural schools often adopts a "shame and blame" approach, contributing to negative body image issues [48]. This approach, focusing on judgment rather than empowerment, may need to be revised to promote a positive body image or address dissatisfaction. Transforming health education paradigms to be more empowering and affirming is crucial for nurturing a healthier perspective on body image.

Limited resources: Limited resources in rural schools pose a significant hurdle in addressing body image issues effectively [48]. Implementing evidence-based interventions or programs that could contribute to students developing a positive body image becomes challenging without adequate resources and expertise. A holistic approach is required to ensure that schools in rural areas receive the necessary support to implement comprehensive and impactful initiatives.

To address the lack of awareness and education on BID in rural areas, it is essential to implement evidence-based interventions and programs focusing on media literacy, self-esteem, and peer influences [12,48]. Additionally, schools and communities should work together to promote a positive body image and challenge the negative messages and images that contribute to BID [47,48].

## Conclusions

In conclusion, this comprehensive review illuminates the intricate dynamics of BID in rural India, exposing a range of factors that shape individual perceptions and societal expectations. From the pervasive influence of cultural norms to the constraints imposed by economic disparities, our exploration underscores the multidimensional nature of BID within rural settings. The identified prevalence across diverse age groups and genders emphasizes the imperative for nuanced interventions that account for these variations. Furthermore, the inextricable link between BID and psychiatric co-morbidities, including depression and anxiety, accentuates the urgency of addressing these intertwined issues within holistic mental health frameworks. Emphasizing the critical importance of tackling BID, this review calls for concerted action from policymakers, healthcare professionals, and communities. Policymakers are urged to integrate culturally sensitive mental health awareness campaigns into public health initiatives and prioritize policies facilitating access to mental health resources in rural areas. Healthcare professionals should have the skills to identify and support individuals experiencing BID, ensuring early intervention. Simultaneously, community leaders and grassroots organizations are encouraged to champion initiatives promoting open dialogues about body image and mental health, creating supportive environments. By collectively addressing BID and its psychiatric co-morbidities, we can foster resilient rural communities that prioritize and celebrate mental well-being. This call to action seeks to initiate positive change, acknowledging challenges and committing to collaborative, culturally informed solutions for a healthier future.

## References

[1] Hosseini SA, Padhy RK (2023). Body image distortion. StatPearls [Internet].

[2] Aparicio-Martinez P, Perea-Moreno AJ, Martinez-Jimenez MP, Redel-Macías MD, Pagliari C, Vaquero-Abellan M (2019). Social media, thin-ideal, body dissatisfaction and disordered eating attitudes: an exploratory analysis. Int J Environ Res Public Health.

[3] Soohinda G, Sampath H, Mishra D, Dutta S (2020). Body image dissatisfaction in young Indian men: prevalence, psychosocial correlates, and the impact of sociocultural pressure. Indian J Soc Psychiatry.

[4] Singh S, Gadiraju P (2020). Prevalence and correlates of body dissatisfaction and disordered eating patterns in Indian youth: the role of media. Indian J Psychiatry.

[5] Karsli Y, Karsli TA (2015). Media effects on body image and eating attitudes of the women living in metropolitan and rural areas in a Turkish population. Procedia Soc Behav Sci.

[6] Nagar I, Virk R (2017). The struggle between the real and ideal: impact of acute media exposure on body image of young Indian women. SAGE Open.

[7] Quittkat HL, Hartmann AS, Düsing R, Buhlmann U, Vocks S (2019). Body dissatisfaction, importance of appearance, and body appreciation in men and women over the lifespan. Front Psychiatry.

[8] Voges MM, Giabbiconi CM, Schöne B, Waldorf M, Hartmann AS, Vocks S (2019). Gender differences in body evaluation: do men show more self-serving double standards than women?. Front Psychol.

[9] Hicks RE, Kenny B, Stevenson S, Vanstone DM (2022). Risk factors in body image dissatisfaction: gender, maladaptive perfectionism, and psychological wellbeing. Heliyon.

[10] Arkenau R, Bauer A, Schneider S, Vocks S (2022). Gender differences in state body satisfaction, affect, and body-related attention patterns towards one’s own and a peer’s body: an eye-tracking Study with Women and Men. Cogn Ther Res.

[11] Lewis-Smith H, Garbett KM, Chaudhry A, Dhillon M, Shroff H, White P, Diedrichs PC (2023). Evaluating a body image school-based intervention in India: a randomized controlled trial. Body Image.

[12] Adithyan GS, Shroff H, Sivakami M, Jacob J (2018). Body image dissatisfaction in adolescents from rural Tamil Nadu, India. Indian J Soc Work.

[13] (2023). Who does it affect?. https://butterfly.org.au/body-image/who-does-it-affect/.

[14] Cafri G, Yamamiya Y, Brannick M, Thompson JK (2005). The influence of sociocultural factors on body image: a meta-analysis. Clin Psychol (New York).

[15] Rymarczyk K (2021). The role of personality traits, sociocultural factors, and body dissatisfaction in anorexia readiness syndrome in women. J Eat Disord.

[16] Azevedo A, Azevedo ÂS (2023). Implications of socio-cultural pressure for a thin body image on avoidance of social interaction and on corrective, compensatory or compulsive shopping behaviour. Int J Environ Res Public Health.

[17] Moy G (2015). Media, Family, and Peer Influence on Children's Body Image (Doctoral dissertation, Rutgers University-Camden Graduate School.

[18] Michael SL, Wentzel K, Elliott MN (2014). Parental and peer factors associated with body image discrepancy among fifth-grade boys and girls. J Youth Adolesc.

[19] Shen J, Chen J, Tang X, Bao S (2022). The effects of media and peers on negative body image among Chinese college students: a chained indirect influence model of appearance comparison and internalization of the thin ideal. J Eat Disord.

[20] (2023). The impact of economic instability on the beauty industry. https://beautymatter.com/articles/the-impact-of-economic-instability-on-beauty.

[21] (2023). The beauty market in 2023: new industry trends. https://www.mckinsey.com/industries/retail/our-insights/the-beauty-market-in-2023-a-special-state-of-fashion-report.

[22] (2023). How do economic factors affect the demand for cosmetics?. https://typeset.io/questions/how-do-economic-factors-affect-the-demand-for-cosmetics-2l9ovfyzy0.

[23] Winter VR, O’Neill E, Cook M (2021). An investigation of socioeconomic status and body image among hook-up app users. J Soc Social Work Res.

[24] O'Dea JA, Caputi P (2001). Association between socioeconomic status, weight, age and gender, and the body image and weight control practices of 6- to 19-year-old children and adolescents. Health Educ Res.

[25] (2023). Research worlds collide to find richer link between body image and income. https://news.virginia.edu/content/research-worlds-collide-find-richer-link-between-body-image-and-income.

[26] von Lengerke T, Mielck A (2012). Body weight dissatisfaction by socioeconomic status among obese, preobese and normal weight women and men: results of the cross-sectional KORA Augsburg S4 population survey. BMC Public Health.

[27] (2023). Malnourishment and poor body image. https://www.eatingdisorderhope.com/information/body-image/malnourishment-and-poor-body-image.

[28] A Rahim NN, Chin YS, Sulaiman N (2019). Socio-demographic factors and body image perception are associated with BMI-for-age among children living in welfare homes in Selangor, Malaysia. Nutrients.

[29] Kim JH (2007). Recognition of body image and food behavior factors among middle school students in San Francisco area. Nutr Res Pract.

[30] Shoraka H, Amirkafi A, Garrusi B (2019). Review of body image and some of contributing factors in Iranian population. Int J Prev Med.

[31] (2023). Body image and physical activity. https://appliedsportpsych.org/resources/health-fitness-resources/body-image-and-physical-activity/.

[32] Services D of H& H (2023). Body image - women. http://www.betterhealth.vic.gov.au/health/healthyliving/body-image-women.

[33] Satghare P, Mahesh MV, Abdin E, Chong SA, Subramaniam M (2019). The relative associations of body image dissatisfaction among psychiatric out-patients in Singapore. Int J Environ Res Public Health.

[34] Barnes M, Abhyankar P, Dimova E, Best C (2020). Associations between body dissatisfaction and self-reported anxiety and depression in otherwise healthy men: a systematic review and meta-analysis. PLoS One.

[35] Edlund K, Johansson F, Lindroth R, Bergman L, Sundberg T, Skillgate E (2022). Body image and compulsive exercise: are there associations with depression among university students?. Eat Weight Disord.

[36] Ziser K, Finklenburg C, Behrens SC (2019). Perceived stress mediates the relationship of body image and depressive symptoms in individuals with obesity. Front Psychiatry.

[37] Pothiraj P, Shamal C, Krishnan V (2022). Body image dissatisfaction, depression, and health-related quality of life amongst indian obese school children: a cross-sectional study. Child Adolesc Ment Health.

[38] Rikani AA, Choudhry Z, Choudhry AM (2013). A critique of the literature on etiology of eating disorders. Ann Neurosci.

[39] Hambleton A, Pepin G, Le A, Maloney D, Touyz S, Maguire S (2022). Psychiatric and medical comorbidities of eating disorders: findings from a rapid review of the literature. J Eat Disord.

[40] Ruffolo JS, Phillips KA, Menard W, Fay C, Weisberg RB (2006). Comorbidity of body dysmorphic disorder and eating disorders: severity of psychopathology and body image disturbance. Int J Eat Disord.

[41] House F (2023). Body image and substance abuse. http://friendlyhousela.org/body-image-and-substance-abuse/.

[42] Lewis-Smith H, Hasan F, Ahuja L, White P, Diedrichs PC (2022). A comic-based body image intervention for adolescents in semi-rural Indian schools: study protocol for a randomized controlled trial. Body Image.

[43] Gaiha SM, Taylor Salisbury T, Koschorke M, Raman U, Petticrew M (2020). Stigma associated with mental health problems among young people in India: a systematic review of magnitude, manifestations and recommendations. BMC Psychiatry.

[44] Kaur A, Kallakuri S, Mukherjee A, Wahid SS, Kohrt BA, Thornicroft G, Maulik PK (2023). Mental health related stigma, service provision and utilization in Northern India: situational analysis. Int J Ment Health Syst.

[45] (2023). Breaking the stigma: addressing mental health in India. http://timesofindia.indiatimes.com/readersblog/myamusings/breaking-the-stigma-addressing-mental-health-in-india-49561/.

[46] Latiff AA, Muhamad J, Rahman RA (2018). Body image dissatisfaction and its determinants among young primary-school adolescents. J Taibah Univ Med Sci.

[47] (2023). How schools contribute to negative body image in kids. http://usnews.com/education/k12/articles/how-schools-contribute-to-negative-body-image-in-kids#:~:text=Body%20size%20is%20a%20common,associated%20with%20weight%20gain%20in.

[48] (2023). How can we protect, promote, and maintain body image?. https://www.mentalhealth.org.uk/our-work/research/body-image-how-we-think-and-feel-about-our-bodies/how-can-we-protect-promote-and-maintain-body-image.

